# Silicon Enhances *Brassica napus* Tolerance to Boron Deficiency by the Remobilisation of Boron and by Changing the Expression of Boron Transporters

**DOI:** 10.3390/plants12132574

**Published:** 2023-07-07

**Authors:** Elise Réthoré, Nusrat Ali, Sylvain Pluchon, Seyed Abdollah Hosseini

**Affiliations:** 1 Plant Nutrition R&D Department, Centre Mondial de l’Innovation of Roullier Group, 35400 Saint Malo, France; elise.rethore@roullier.com (E.R.); sylvain.pluchon@roullier.com (S.P.); 2 Phys-Chem and Bio-Analytics R&D Department, Centre Mondial de l’Innovation of Roullier Group, 35400 Saint-Malo, France; nusrat.ali@roullier.com

**Keywords:** nutrient crosstalk, nutrient deprivation, plant nutrition, gene regulatory network, stress tolerance

## Abstract

Boron (B) is an essential micronutrient for plants, and its deficiency is a widespread nutritional disorder, particularly in high-demanding crops like *Brassica napus*. Over the past few decades, silicon (Si) has been shown to mitigate plant nutrient deficiencies of different macro- and micro-nutrients. However, the work on B and Si cross-talk has mostly been focused on the alleviation of B toxicity by Si application. In the present study, we investigated the effect of Si application on rapeseed plants grown hydroponically under long-term B deficiency (20 days at 0.1 µM B). In addition, a B-uptake labelling experiment was conducted, and the expression of the genes involved in B uptake were monitored between 2 and 15 days of B shortage. The results showed that Si significantly improved rapeseed plant growth under B deficiency by 34% and 49% in shoots and roots, respectively. It also increased the expression level of *BnaNIP5;1* and *BOR1;2c* in both young leaves and roots. The uptake labelling experiment showed the remobilization of previously fixed ^11^B from old leaves to new tissues. This study provides additional evidence of the beneficial effects of Si under conditions lacking B by changing the expression of the *BnaNIP5;1* gene and by remobilizing ^11^B to young tissues.

## 1. Introduction

Rapeseed (*Brassica napus*) is the most important oil crop grown in Europe and the third most important in the world after soybean and palm oil [1,2]. It is used for animal and human nutrition, as well as for industrial purposes such as biofuel production [3]. To ensure its growth, rapeseed requires high levels of nutrients, particularly nitrogen, sulfur and boron (B) [4,5,6]. The essentiality of the microelement B was first evidenced in 1923 by Warington [7]. During crop development, it is involved in shoot and root meristem activity, in the stimulation of the reproductive stage (particularly the sensitive stages of microspore development and during pollen germination) and in the improvement of seed quality [8,9]. At the molecular level, it plays an important role in the structure of cell walls and membrane function [9,10]. Indeed, about 80–90% of B is located in the cell wall, where borate is cross-linked with rhamnogalacturonan II dimers to strengthen the cell wall structure [11,12]. Moreover, B is involved in sugar transport, nucleic acid synthesis, nitrogen and phenolic metabolism and indole acetic acid (IAA) metabolism [9].

*Brassica napus* cultivars have soil B concentration requirements higher than 0.5 mg B (kg soil)^−1^ [13]. B is mainly present in the form of boric acid (H_3_BO_3_) and borate anion [B(OH)_4_^−^] in the soil and in plants [9,10]. It is mostly absorbed through mass flow, and it was thought to be exclusively transported through the plasma membrane by a passive diffusion mechanism. However, B transporters have been identified in roots, shoots and reproductive organs and were shown to respond to the level of B in the soil solution [10]. Two major types of transporters are involved in the regulation of B uptake: the boric acid channel NIPs (Nodulin26-like intrinsic proteins) and the B exporters, BORs [14]. Under B limitation, NIP5;1 and BOR1 would be the major transporters for B uptake in the root and its translocation to the shoot via xylem loading, respectively [15,16]. Overexpression of these transporters in *Arabidopsis thaliana* led to increased B deficiency tolerance [17,18].

The response of crops to B deficiency has been investigated over the past few decades. The reproductive stage is known to be particularly sensitive to B shortage, since the requirements of B are higher during this developmental stage [19]. However, at the vegetative stage, B deficiency also causes serious problems for proper plant development. Indeed, B deficiency was shown to inhibit root growth and development by interfering with IAA metabolism and transport [20]. It also impacts the architecture of the shoot by inhibiting the growth of the apex and the apical dominance [21]. Moreover, B deficiency can also disturb the plant water relations by reducing root growth, damaging the xylem structure or altering cuticle or stomata function, which can impact the plant response to abiotic stresses such as drought or cold [22]. Although the genetic selection of more B-efficient crops is ongoing [5], other means of improving B nutrition should also be considered.

Silicon (Si) is recognized as a non-essential but beneficial element for plants [23]. Indeed, even though Si is not required for the growth of most crops, its positive effect on the plant response to diverse biotic and abiotic stresses and nutritional disorders has been highlighted over the past decades [24,25,26,27,28]. Most studies focused on the characterization of Si-accumulating plants like rice or cucumber [29,30,31,32,33,34]. However, some studies also highlighted the beneficial effect of Si in non-accumulating plants exposed to nutrient deficiency [35,36,37]. For example, in rapeseed, Si has been shown to alleviate the effect of nitrogen deficiency by delaying leaf senescence and promoting the expression of N transporters [38]. Nevertheless, in a recent study, no clear positive influence of Si could be observed in rapeseed plants under S deficiency: Si supply did not increase the biomass or S content in these conditions [39]. Regarding B and Si interaction, most of the work has been focused on the effect of Si in alleviating B toxicity [40,41,42,43,44,45]. For instance, the work done by Inal et al. (2009) demonstrated that the application of Si increased the level of non-enzymatic antioxidant activity and the activities of major antioxidant enzymes like superoxide dismutase, catalase and ascorbate peroxidase in barley plants under B toxicity [41]. Other relevant studies have also reported the regulatory role of Si in alleviating B toxicity, mainly by reducing osmotic stress and by modulating the activities of antioxidant enzymes and the reduction in leaf B accumulation [42,46]. However, the role of Si under B deficiency has been rarely investigated. It was shown that foliar or root application of Si was able to mitigate boron deficiency in cotton plants by improving the chlorophyll content and photosynthetic performance [47]. In addition, in rapeseed, Si improved the growth and B uptake under low-B conditions, but the molecular mechanisms involved in this process were not studied [48]. Therefore, in the present work, we studied the cross-talk between B and Si nutrition in two different experiments. In the first experiment, the response of rapeseed to B deficiency and Si application was considered under long-term conditions (20 days). In the second experiment, we monitored the B uptake both in the short and long periods of B deprivation (2 to 15 days) using an uptake labelling experiment and by monitoring the expression of B transporters in roots and shoots.

## 2. Results

### 2.1. Silicon Application Promotes Plant Growth under Long-Term B Deficiency

**Figure 1:**
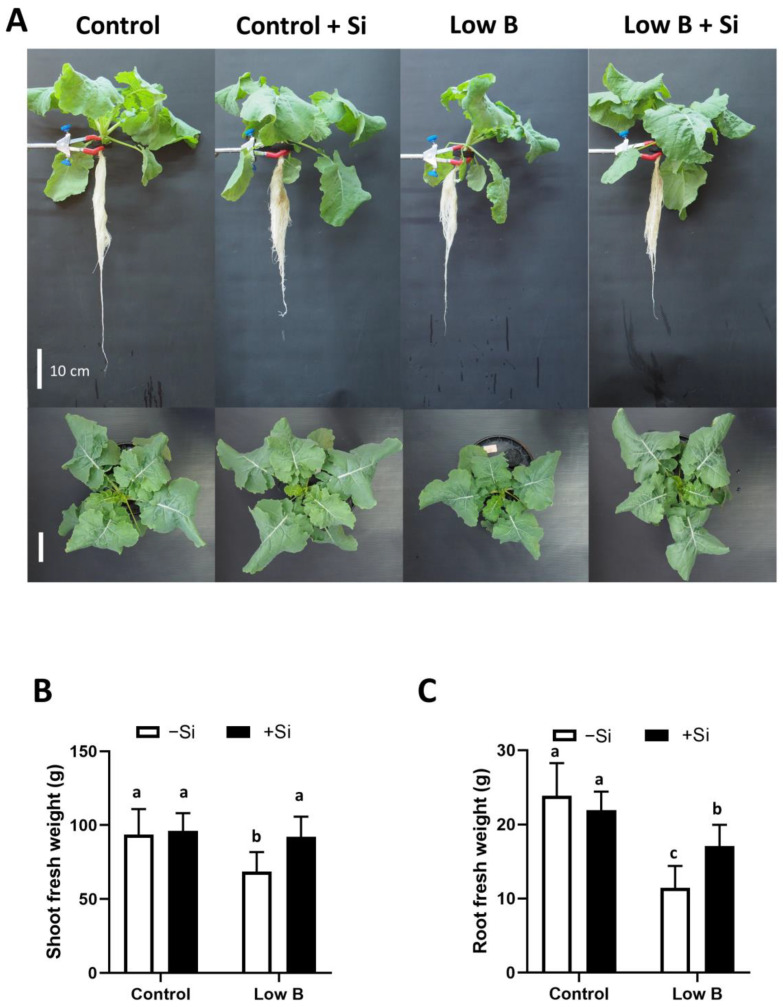
Effect of Si application on root and shoot biomass of rapeseed under long-term B deficiency. (**A**) Rapeseed phenotype under ample and deficient B supply and Si application, (**B**) shoot fresh weight and (**C**) root fresh weight. Plants were grown in hydroponic culture under either low (0.1 µM) or optimal B (25 µM) as well as a supply of 1.8 mM Si. Roots and shoots were harvested after 20 days of treatment. Bars indicate mean ± SD. Different letters denote significant differences according to the ANOVA followed by the SNK test (*p* < 0.05; *n* = 6).

The response of rapeseed to B deficiency and Si application was first assessed under long term B deficiency after 20 days of stress application (Figure 1A). The effect of B deficiency was visible on both shoot and root fresh weight with a significant decrease of 27% and 52%, respectively (Figure 1B,C). We did not observe any significant effect in plants grown under normal B levels after the application of Si; however, under low B, the application of Si significantly increased shoot and root fresh weight by 34% and 49%, respectively (Figure 1B,C). Notably, the shoot fresh weight even recovered to the level of control plants under conditions lacking B when plants did receive Si (Figure 1B). Two-way ANOVA revealed that there was a significant interaction between B and Si for root biomass (F-value = 7.031, Pr(>F) = 0.0174).

**Figure 2:**
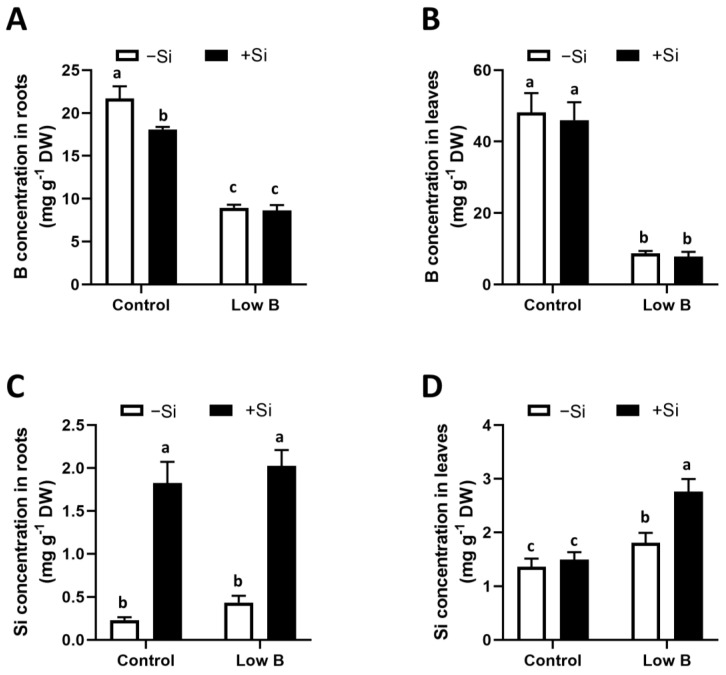
Effect of Si application on root and shoot B and Si concentrations in rapeseed under B deficiency. (**A**) B concentration in roots, (**B**) B concentration in leaves, (**C**) Si concentration in roots and (**D**) Si concentration in leaves. Plants were grown in hydroponic culture under either low (0.1 µM) or optimal B (25 µM) as well as a supply of 1.8 mM Si. Roots and shoots were harvested after 20 days of treatment. Bars indicate mean ± SD. Different letters denote significant differences according to the ANOVA followed by the SNK test (*p* < 0.05; *n* = 6).

We further performed an elemental analysis to assess the concentration of both B and Si (Figure 2). As expected, the concentration of B was much lower in both the roots and leaves of B-deficient rapeseed plants compared with control plants, which further validated the experimental setup. Under control conditions, Si significantly decreased the B concentration in the roots. In addition, Si supply did not show any effect under low-B conditions in both organs (Figure 2A,B). Looking at the Si concentration, except for control conditions in the leaves, the application of Si significantly increased its level in both the roots and leaves when Si was provided (Figure 2C,D). A significant interaction between B and Si was also detected by the two-way ANOVA for Si content in leaves (F-value = 23.07, Pr(>F) = 0.000195). We have also analysed the concentrations of other nutrients in the present work, and the results are presented in Supplementary Table S2. In roots, B deficiency induced a decrease in the concentration of the macro-elements Ca and Mg and in the micro-elements Al and Cu, while in leaves, it induced an increase in Ca concentration and a decrease in Zn concentration. Meanwhile, Si application induced a decrease in Ca, P, Al, Cu, Fe and Zn levels in roots under control conditions. In shoots, it could enhance the K concentration in the leaves but decreased the Zn concentration. In brief, compared with the low-B condition, the application of Si displayed a decrease in the levels of P, Fe and Zn in the roots without any significant changes in the leaves (Supplementary Table S2A). To check whether these observations were due to a dilution effect, we also calculated the total uptake per plant in the roots and shoots by multiplying the concentration of each element by the dry weight of the organs (Supplementary Table S2B). In general, we observe similar patterns between concentration and total uptake, meaning that the differences in the concentrations are not directly correlated with the difference in growth observed (Supplementary Table S2B).

### 2.2. Silicon Application Promotes the Growth of New Leaves and the Expression of B Transporters under Short-Term B Deficiency

**Figure 3:**
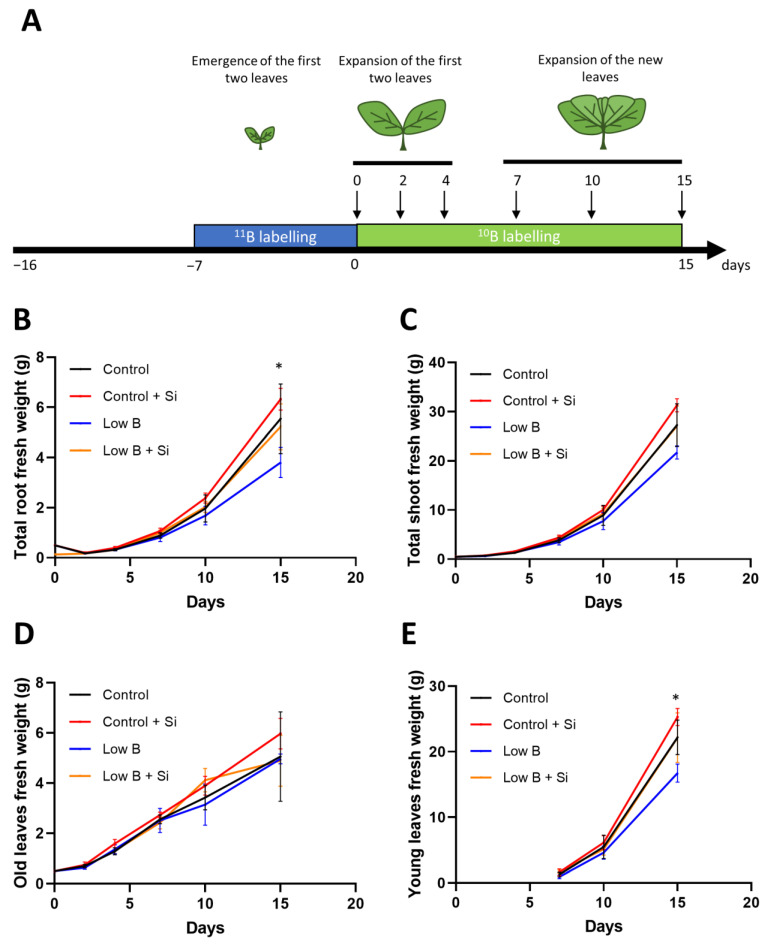
Effect of Si application on root and shoot biomass of rapeseed under short and long-term B deficiency. (**A**) Scheme of the uptake labelling experiment. (**B**) Total root fresh weight. (**C**) Total shoot fresh weight. (**D**) Old leaves fresh weight. (**E**) New leaves fresh weight. Plants were grown in hydroponic culture under optimal B conditions for one week (using 25 µM ^11^B) and then for two weeks under either low or optimal B (0.1 µM or 25 µM ^10^B, respectively) as well as a supply of 1.8 mM Si. Roots and shoots were harvested after 0, 2, 4, 7, 10 and 15 days of treatment. The first two developed leaves are considered “old leaves” and the other leaves developing from 7 days of B deficiency are considered “young leaves”. Bars indicate mean ± SD. Asterisks denote a significant difference between low B and the other modalities (*p* < 0.05; *n* = 5) according to the ANOVA followed by the SNK test.

The positive effect of Si on the growth of rapeseed under conditions lacking B was obvious in the first experiment; however, no effect of Si could be observed on the B status under long-term deficiency. Hence, a second experiment was performed using an uptake labelling method to evaluate the effect of Si on the early response of rapeseed plants to B deficiency and to examine if Si could influence the root to shoot translocation of B and mainly from old to newly emerged leaves. For this purpose, rapeseed seedlings were grown under 25 µM ^11^B-enriched boric acid for one week and then transferred to 0.1 µM or 25 µM ^10^B and harvested at different timepoints after transfer (0, 2, 4, 7, 10 and 15 days) (Figure 3A). The fresh weight evolution of the organs was examined considering the two first-developed leaves as “old leaves” and the rest of the leaves as “young leaves”. Interestingly, the effect of B deprivation on the total root and young leaf shoot biomass was significant from 15 days after the start of low-B application, whereas Si application maintained their values close to control plants (Figure 3B,E). However, we did not observe a significant effect of Si on the total shoot fresh weight of old leaves under low-B conditions (Figure 3C,D).

**Figure 4:**
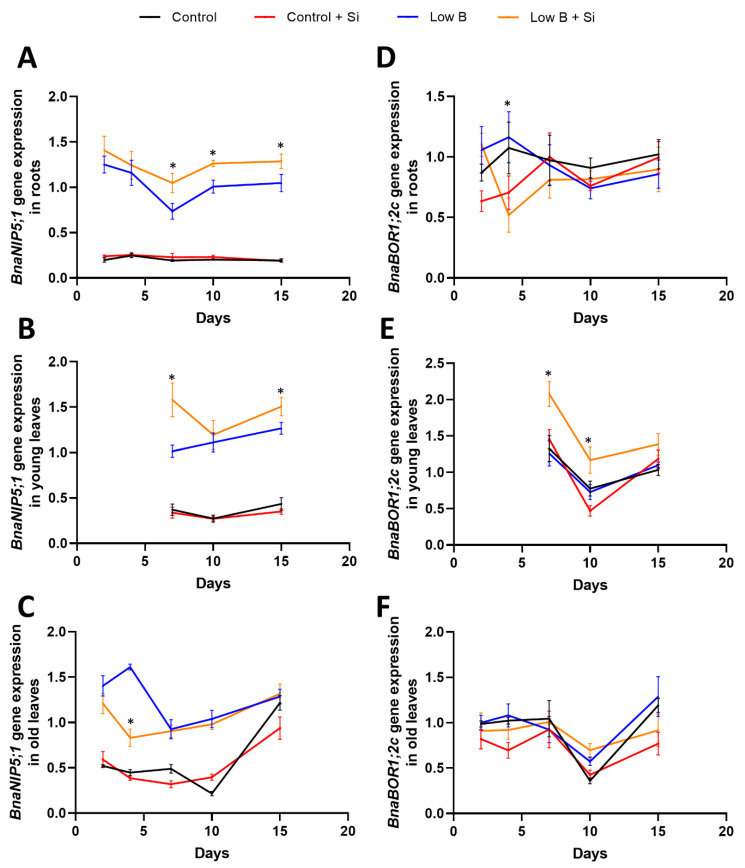
Effect of Si application on the expression level of the genes involved in B transporters in rapeseed under short-term B deficiency. (**A**) Relative expression of *BnaNIP5;1* in roots. (**B**) Relative expression of *BnaNIP5;1* in young leaves. (**C**) Relative expression of *BnaNIP5;1* in old leaves. (**D**) Relative expression of *BnaBOR1;2c* in roots. (**E**) Relative expression of *BnaBOR1;2c* in young leaves. (**F**) Relative expression of *BnaBOR1;2c* in old leaves. Plants were grown in hydroponic culture under optimal B conditions for one week (using 25 µM ^11^B) and then for two weeks under either low or optimal B (0.1 µM or 25 µM ^10^B, respectively) as well as a supply of 1.8 mM Si. Roots and shoots were harvested after 0, 2, 4, 7, 10 and 15 days of treatments. The first two developed leaves are considered “old leaves” and the other leaves developing from 7 days of B deficiency are considered “young” leaves. Bars indicate mean ± SEM. Asterisks denote a significant difference between low B and low B+Si (*p* < 0.05; *n* = 5) according to the *t*-test.

To observe closely the early modulations of B deprivation in different tissues, we analysed the expression pattern of genes encoding Nodulin26-like Intrinsic Proteins (NIPs) and *BOR1* transporters, which are known to regulate the uptake and translocation of B in *Brassica napus*. Among the two NIP genes (*BnaNIP5;1* and *BnaNIP6;1*) and six BOR1 genes (*BnaBOR1;1a*, *BnaBOR1;1c*, *BnaBOR1;2a*, *BnaBOR1;2c*, *BnaBOR1;3a*, *BnaBOR1;3c*) analysed, we could observe significant changes in the expression of selected genes in different tissues (Figure 4 and Figures S1–S3). Specifically, under low B, induction of *BnaNIP5;1* expression was noticed in roots, old leaves and young leaves in comparison with control plants that did receive sufficient B (Figure 4A–C). This induction was significantly pronounced under low B+Si compared with low B in roots and young leaves (Figure 4A,B). Two-way ANOVA (*p* < 0.05) showed a significant interaction between B and Si for *BnaNIP5;1* gene expression at all timepoints harvest in both the roots and young leaves but not in old leaves. Among the BOR1 transporters, *BnaBOR1;2c* was significantly induced in the young leaves under low B+Si compared with sole B deficiency, while their expression levels followed a similar pattern across different time points in all treatments (Figure 4E). Indeed, a significant interaction between B and Si according to two-way ANOVA (*p* < 0.05) was detected for *BnaBOR1;2c* gene expression at all timepoints in young leaves, but not in roots or old leaves. Another *BOR1* family gene, *BnaBOR1;3c*, displayed a contrasting pattern across different harvest times in roots under low B+Si compared with other treatments and showed significantly higher expression levels under low B+Si compared with sole B deficiency after 10 and 15 days of imposing the B shortage (Supplementary Figure S1E). In old leaves, all the BOR1 transporters (*BnaBOR1;2a*, *BnaBOR1;3a*, *BnaBOR1;3c*) and *BnaNIP6;1* showed induction four days after low-B treatment compared with all other treatments (Supplementary Figure S2).

### 2.3. Silicon Improves ^11^B Remobilization from Old to Young Tissues

**Figure 5:**
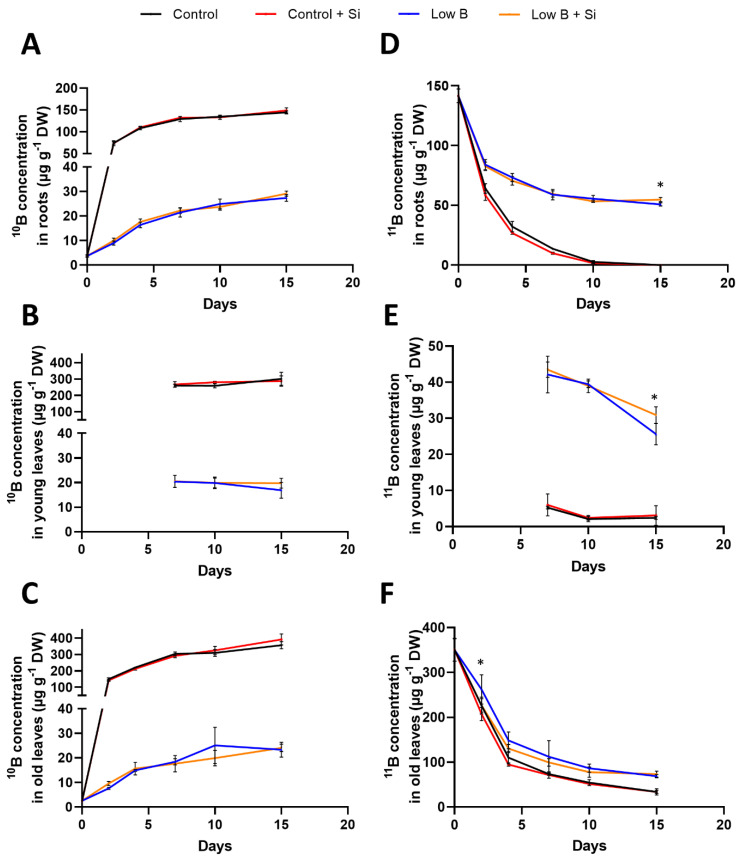
Uptake labelling experiment in B-deficient rapeseed plants treated with Si. (**A**) ^10^B concentration in roots, (**B**) ^10^B concentration in young leaves, (**C**) ^10^B concentration in old leaves, (**D**) ^11^B concentration in roots, (**E**) ^11^B concentration in young leaves and (**F**) ^11^B concentration in old leaves. Plants were grown in hydroponic culture under control B conditions for one week (using 25 µM ^11^B) and then for two weeks under either low or optimal B (0.1 µM or 25 µM ^10^B, respectively) as well as a supply of 1.8 mM Si. Roots and shoots were harvested after 0, 2, 4, 7, 10 and 15 days of treatment. The first two developed leaves are considered “old leaves” and the other leaves developing from 7 days under B deficiency are considered “young leaves”. Bars indicate mean ± SD. Asterisks denote a significant difference between low B and low B+Si (*p* < 0.05; *n* = 5) according to the *t*-test.

The same samples that were used for gene expression analysis were analysed by ICP-MS to determine their concentration of ^10^B and ^11^B in the different organs (roots, young leaves and old leaves). As expected, under control conditions, the concentration of ^10^B increased over time, while the concentration of ^11^B decreased in all organs (Figure 5). No clear effect of Si application under control conditions was visible. Regarding the low-B plants, the concentration of ^10^B was obviously much lower compared with control plants in each organ (Figure 5A–C) and that of ^11^B was maintained at a higher level, particularly in roots and young leaves (Figure 5D–F). No significant effect on ^10^B concentration was visible in the different organs under low B+Si conditions compared with the low-B condition. Nevertheless, a significant effect of Si application under B deficiency was visible for the ^11^B concentration both in roots and young leaves but not in old leaves when conditions lacking B was extended further to 15 days. On the contrary, a significant decrease in the ^11^B concentration in old leaves was observed at two days after B deficiency by Si application (Figure 5F). These data suggest that Si would favour ^11^B translocation from old to young tissues under B deficiency.

## 3. Discussion

One of the most noticeable properties of Si nutrition is its considerable role in alleviating environmental stresses. During the last decade, the effect of Si on the mitigation of abiotic and biotic stresses has been put forth in the literature [25,27,49], wherein drought and salinity were the two most frequent stress triggers. Within abiotic stresses, a conclusive role of Si in the alleviation of individual mineral nutrient deficiencies has also been demonstrated in major scientific articles [26,35,50]. With the exception of iron, which is considered a deficient element, micronutrients are mainly investigated as toxic elements with regard to Si nutrition.

In this regard, researchers have assessed the regulatory role of Si under an excess of B mainly in soil-grown cereals [40,41,46], and the role of Si under B deficiency has been rarely explored.

Rapeseed is a high nutrient-demanding crop, and the importance of nitrogen and B for its growth and development has been well discussed in different literature reports [2,5,38,51]. In the present work, we aimed to decipher the cross-talk between Si and B nutrition in the Si-non-accumulating plant. In our first experiment, we showed a positive and significant response of rapeseed plants to Si nutrition at the biomass level both for roots and shoots under conditions lacking B. The positive effect of Si at the biomass level was also already shown under different nutrient deficiencies such as nitrogen [38], potassium [52], magnesium [53], sulfur [34] and iron [54,55,56]. The increases in the biomass of rapeseed by the application of Si under B deprivation was accompanied by a significantly higher accumulation of Si in both roots and shoots without any changes in B concentration. This further encouraged us to investigate whether Si modulates the expression of B transporters and contributes to the uptake and translocation of B under the lack of B nutrition. The uptake labelling experiment showed a higher concentration of ^11^B compared with ^10^B under B deprivation, which could be explained by the previous acquisition of ^11^B before low ^10^B application. In low B+Si plants, the uptake of ^10^B was not significantly increased compared with low-B plants, but the ^11^B concentration in the roots and young leaves of B-deficient plants increased after 15 days of Si application. This reflects a higher remobilization of ^11^B from old to young tissues after Si application, which could maintain plant growth despite the lack of B.

We also monitored the changes in the expression levels of B transporters. There are two major sophisticated pathways for B uptake and translocation. In brief, the passive bidirectional Nodulin26-like Intrinsic Protein (NIP) channels facilitate the diffusion of boric acid across membranes. Then, the active efflux transporters, BORs, transport the borate anion [14,57]. NIPs belong to the Major Intrinsic Protein family, which comprises distinct subfamilies being either essential for the regulation of the plant water homeostasis or for the facilitated diffusion of small solutes, including metalloid acids [58,59], while BORs function mainly either in the active transfer of B to neighbouring cell types or in the removal of B from cells into the apoplast to confer tolerance to high B [14]. These two transporters are often co-expressed in the same cell while trafficking to opposite sides of the cell, thus functioning synergistically to optimize transcellular B fluxes and to actively generate and maintain B gradients [60].

*Brassica napus* is very sensitive to the lack of B and exhibits detrimental and irreversible B-deficiency symptoms, such as “root rot” or “flowering without seed setting”, and yield losses [61]. The role and function of both NIPs and BOR transporters were investigated in rapeseed plants. Diehn et al., 2019, showed the expression of specific sets of functional Nodulin26-like Intrinsic Proteins and BOR1 transporters in *Brassica napus* [57]. Interestingly, in the present work, Si increased the expression of *BnaNIP5;1* after 7 days of B deficiency in roots and young leaves. We observed a similar change in the expression of the *BnaBOR1;2C* transporter that was mainly induced in young leaves after 7 days of B shortage. The changes in the *BnaNIP5;1* gene were in agreement with the findings of Diehn et al., 2019. These authors suggested that the NIP5 family are the key players in the B fluxes throughout the plant and highlighted the importance of both NIPs and BOR genes for the fertility of plants under B deprivation.

In addition, the changes in the expression of *BnaBOR1;2 C* was in line with another study in rapeseed, in which Chen et al. (2018) showed the diversity and potential of BOR family (BOR1;1C) members in improving B nutrition [62]. Therefore, we believe that Si mitigates B deficiency in rapeseed by changing primarily the expression of *BnaNIP1;5* in roots and young leaves and the regulation of the *BnaBOR1;2C* gene in young leaves, which could be related to higher ^11^B remobilization to these organs.

Notably, in our research study, the higher expression level of *BnaNIP1;5* with Si supply coincided with a higher root biomass. In fact, it was previously shown that the overexpression of *BOR1* under the control of the cauliflower mosaic virus 35S RNA (CaMV 35S) promoter resulted in improved root growth and fertility under B-deficient conditions [17]. We have already shown that supplementation with Si improves the root growth in rice under S deficiency or in barley under K deficiency [34,63]. In the present work, we could show that Si positively regulates B homeostasis in rapeseed plants under conditions lacking B by increasing root growth and probably by improving B translocation through the regulation of *BnaNIP1;5* gene expression.

## 4. Material and Methods

### 4.1. Plant Material and Growth Conditions

The hydroponic experiments were conducted in a greenhouse equipped with high-pressure sodium lights (60% humidity, 16 h days at 21 °C and 8 h nights at 18 °C). Rapeseed seeds (cv Trezzor) were germinated for 9 days on vermiculite and were then transplanted to 5 L opaque pots with 0.5× Hoagland solution, each containing 3 plants. Six replicates per modality were performed. The hydroponic solution was buffered to pH 5.9 and renewed two times a week with full Hoagland solution (2.5 mM Ca(NO_3_)_2_, 2 mM KCl, 1 mM MgSO_4_, 0.5 mM NH_4_H_2_PO_4_, 0.5 mM CaCl_2_, 25 µM H_3_BO_3_, 5 µM MnSO_4_, 0.8 µM ZnSO_4_, 0.3 µM CuSO_4_, 0.1 µM (NH_4_)_6_Mo_7_O_24_ and 100 µM EDTA,2NaFe) with continuous aeration. After one renewal of the solution with full Hoagland solution, B deprivation was started six days after transplanting in B-sufficient conditions by reducing the B concentration to 0.1 µM in the medium. Monosilicic acid [Si(OH)_4_] was prepared by passing a sodium silicate solution through a column filled with cation-exchange resins (Amberlite, Sigma Aldrich, St. Louis, MO, USA) according to [64]. A quantity of 100 g of sodium silicate was dissolved in 5 L and passed slowly through a previously hydrated column containing 900 g of Amberlite^®^ IRC120 H, hydrogen form (Sigma Aldrich). The concentration of Na and Si was then measured by inductively coupled plasma-optical emission spectrometry (see paragraph below) to check the purity of the solution. An aliquot of 1.8 mM Si was provided for plants at the beginning of the B deficiency and each time the solution was renewed. Samples were harvested after 20 days of B deficiency and the total fresh weight of the shoots and the roots was determined. The fifth and sixth leaves and the roots were harvested for elemental analysis.

### 4.2. Elemental Analysis

Elemental analysis was performed using inductively coupled plasma-optical emission spectrometry (ICP-OES, 5110 VDV, Agilent, Santa Clara, CA, USA) with prior microwave acid sample digestion (Multiwave Pro, Anton Paar, Les Ulis, France) (8 mL of concentrated HNO_3_, 2 mL of H_2_O_2_ and 15 mL of Milli-Q water per 100 mg dry weight (DW)) [65]. The quantification of each element was carried out with an external standard calibration curve. For Si determination, the high-temperature alkaline fusion method was adapted from [66] using Si standard solution (Merck, Darmstadt, Germany). A volume of 1.5 mL of 50% NaOH was added to 50 mg of samples (DW) and mixed well in autoclavable tubes. The sample tubes were autoclaved for 20 min at 121 °C. The solution was then transferred to volumetric flasks and the volume was made up to 5 mL with distilled water. For the subsequent reaction, 160 µL of the sample solution was transferred to a 5 mL Eppendorf tube and 1.2 mL of 20% acetic acid followed by 400 µL of ammonium molybdate solution (54 g/L, pH 7.0) was added. The reaction mixture was mixed thoroughly and kept for 5 min at ambient temperature. Next, 200 µL of 20% tartaric acid and 40 µL of reducing solution (containing 63.5 mM Na_2_SO_3_, 0.96 M NaHSO_3_ and 6.7 mM 1-amino-2-naphthol-4-sulfonic acid) were added. Thirty minutes later, the absorbance was measured at 650 nm.

### 4.3. Boron Uptake Labelling and Translocation

In order to better characterize the response of rapeseed to short-term B deficiency, an uptake labelling experiment was conducted. After germination on vermiculite, rapeseed seedlings were transplanted in Hoagland solution containing 25 µM ^11^B (H_3_^11^BO_3_, 99%, Cambridge Isotope Laboratories, Andover, MA, USA) for one week and were then transferred to control (25 µM) or B-deficient conditions (0.1 µM) containing only ^10^B (H_3_^10^BO_3_, 99%, Cambridge Isotope Laboratories) in the presence or absence of 1.8 mM Si (Figure 3A). Plants were harvested at 0, 2, 4, 7, 10 and 15 days after ^10^B and Si application for B analysis and gene expression analysis. For the harvests at 0, 2 and 4 days, only the first two leaves which had previously received ^11^B were developed, so the whole shoot was harvested. For the harvests at 7, 10 and 15 days, new leaves which had never received ^11^B (referred to as “young leaves”) were harvested separately from the first two leaves (referred to as “old leaves”).

Rapeseed shoot and root (10 mg dry weight) were weighed into PTFE digestion tubes. Concentrated nitric acid (0.5 mL) was added to each tube. Samples were digested under pressure using a high-performance microwave reactor (Ultraclave 4; MLS, Germany). Digested samples were transferred to Greiner centrifuge tubes and diluted with de-ionized Milli-Q water to a final volume of 8 mL [67]. Elemental analysis was carried out by inductively coupled plasma mass spectrometry (ICP-MS) using the sector-field high-resolution (HR)-ICP-MS (ELEMENT 2, Thermo Fisher Scientific, Dreieich, Germany) with software version 3.1.7.278. An external calibration curve was set at a concentration range from 1 ppb to 640 ppb. Boron isotope calibration solutions were prepared from boric acid standards ^10^B, 99% (ref. BLM-870-5) and ^11^B, 99% (ref. BLM-797-5) (Cambridge Isotope Laboratories, Inc., Tewksbury, MA, USA). The element rhodium (Rh) was infused inline and used as the internal standard for matrix correction.

### 4.4. RNA Extraction and Gene Expression Analysis

The root and leaf samples (100 and 70 mg, respectively) of rapeseed plants harvested for each time point (2, 4, 7, 10 and 15 days after ^10^B and Si application) were ground to a fine powder in the presence of liquid nitrogen, and the total RNA was extracted using a Nucleospin 8 RNA kit (Macherey-Nagel, Düren, Germany) following the protocol of the manufacturer. The quality and yield of all RNA samples were analysed and checked in a 4200 Tapestation (Agilent Technologies, Santa Clara, CA, USA) followed by DNase treatment and complementary DNA (cDNA) synthesis from 1 µg RNA using an iScript genomic DNA (gDNA) clear cDNA synthesis kit (Bio-Rad, Hercules, CA, USA). Quantitative RT-PCR (qPCR) analysis was performed in a total volume of 10 µL using the Universal SYBR Green Supermix (Bio-Rad) in an RT PCR Detection System (Bio-Rad). The qPCR reactions were performed in technical triplicates using independent cDNA reactions for each biological replicate and 300 nM gene-specific primer pairs. Specific primers for all candidate genes were designed using Primer3 software (version 0.4.0) and are listed in Supplementary Table S1. The thermal cycler protocol included 98 °C for 3 min, 40 cycles of 98 °C for 15 s, 60 °C for 30 s, 72 °C for 15 s, and a final 3 min extension at 72 °C. The expression of all the candidate genes was normalized against four rapeseed reference genes, namely *BnaEf1alpha*, *BnaACT7*, *BnaTIP41* and *BnaPP2A*. The selection of the four reference genes was based on the published literature comparing reference gene stability in stress conditions in *Brassica* species [68,69]. We analysed the four reference genes in both the roots and the leaves of the experimental samples and used the inbuilt Reference Gene Selection tool provided with CFX Maestro software version 1.0 (Bio-Rad) to evaluate the stability of these reference genes in all the tested samples. Normalized expression of each sample for selected candidate genes was calculated using CFX Maestro software version 2.2 (Bio-Rad). Statistics were performed for each time point independently.

### 4.5. Statistical Analysis

Data are represented as the mean ± standard deviation (SD) or standard error of the mean (SEM) for *n* = 5 or 6. The analysis of variance (ANOVA) and the post-hoc Student–Newman–Keuls (SNK) (R software) were employed to analyse the data, and these were marked by different letters when significantly different (*p* < 0.05). Two-way ANOVA was also performed on the different measured parameters to detect the interaction between Si and B treatments.

## 5. Conclusions

The present study provides further evidence that Si nutrition mitigates B deficiency in non-accumulator rapeseed plants. Si could increase biomass production under conditions lacking B, which coincided with a higher accumulation of Si in roots and leaves. The application of Si under conditions lacking B was also shown to increase the expression level of *BnaNIP5;1* in both roots and young leaves and, at the same time, to increase the transcript level of *BnaBOR1;2c* in young leaves. Thus, we believe that the changes in the level of *BnaNIP5;1* and its correlation with a higher root biomass and a better translocation of B from old to young tissues contribute to the tolerance of rapeseed plants to B deprivation.

## Data Availability

All data are available in the manuscript file.

## Supplementary Materials

The following supporting information can be downloaded at: https://www.mdpi.com/article/10.3390/plants12132574/s1, Figure S1: Effect of Si application on the expression level of B transporters in rapeseed roots under short-term B deficiency; Figure S2: Effect of Si application on the expression level of B transporters in old rapeseed leaves under short-term B deficiency; Figure S3: Effect of Si application on the expression level of B transporters in young rapeseed leaves under short-term B deficiency; Table S1: List of primers used for gene expression analysis; Table S2: Effect of Si application on rapeseed elemental composition under B deficiency.
