# Supplementary material for: Silicon Enhances Brassica napus Tolerance to Boron Deficiency by the Remobilisation of Boron and by Changing the Expression of Boron Transporters
Source: Plants (Basel). 2023 Jul 7;12(13):2574. doi: 10.3390/plants12132574 (PMC10346523; doi:10.3390/plants12132574)

Supplementary Table S1. List of primers used for gene expression analysis.

GENE	ACCESSION NO.	FORWARD PRIMER	REVERSE PRIMER	AMPLICON SIZE (BP)
BnaNIP5;1	KT899999.1	5'-CTCCTGGTGTCTCCCTTACTC-3'	5'-GCTCCGTCGTATTTCTGGTTC-3'	109
BnaNIP6;1	XM_013794870.3	5'-TGGAGGTTTTGAAGGAAAGAGG-3'	5'-CGCAAGAGACTGGAGGGA-3'	134
BnaBOR1;1a	GU827643	5'-AAAGAGCAGCGTGTAAGCAA-3'	5'-CAGAGGACAGAGGTTGGGA-3'	98
BnaBOR1;1c	GU827644	5'-CAAAGAGCAGCGTGTAAGCAA-3'	5'-GCGAAGTAGCCCCAAAGGA-3'	111
BnaBOR1;2a	GU827648	5'-CGGAACGGGAGAATCAGAAG-3'	5'-AAGACCAAAGGGAGGAACCA-3'	89
BnaBOR1;2c	GU827647	5'-TAGACCTGAACTGGGACGAGA-3'	5'-GCCATCACAAACAGCATCAAAG-3'	84
BnaBOR1;3a	GU827646	5'-AATGGTGGGAGGATGCGTTG-3'	5'-CGACTTGGGGCTGTGAATAGA-3'	150
BnaBOR1;3c	GU827645	5'-CACTGCTTATTCTCGGCGTT-3'	5'-AGTCCAAACACAAACCCATCC-3'	131
BnaEf1alpha	NM_001315606	5'-AGTCTCTTGTGGAGGCACTT-3'	5'-CTGGGTCATCCTTGGGGTT-3'	111
BnaACT7	NM_001316079	5'-TGAGAGATTCCGTTGCCCT-3'	5'-CACCACTGAGGACGATGTTTC-3'	149
BnaTIP41	XM_013842392	5'-GGCACGATTCTCACTTCTCTC-3'	5'-CATTCTCGCCAAAGACCATTTC-3'	88
BnaPP2A	XM_013856328	5'-TCCTTTTGTTGGAGGGGTTG-3'	5'-CTCTCTCACGCAGGTCTCTT-3'	98

Supplementary Table S2 : Effect of Si application on rapeseed elemental composition under B deficiency. Plants were grown in hydroponic culture under either low (0.1 µM) or normal B (25 µM) as well as 1.8 mM of Si supply. Roots and shoots were harvested after 20 days of treatments. Data are expressed as mean ± SD in mg g^{-1} dry weight for the concentration (A) or in mg per plant for the total uptake (B). Data expressed as mg per plant correspond to the concentration in root or shoot multiplied by the corresponding root or shoot dry weight. Different letters denote significant difference according to ANOVA followed by the SNK test ($p < 0.05$; n = 6).

(A)

	Roots (mg g^{-1} DW)				Leaves (mg g^{-1} DW)			
	Control		Low B		Control		Low B	
	-Si	+Si	-Si	+Si	-Si	+Si	-Si	+Si
**Al**	0.11 ± 0.02 ^{a}	0.04 ± 0.01 ^{c}	0.07 ± 0.01 ^{b}	0.06 ± 0.02 ^{bc}	0.04 ± 0.00	0.04 ± 0.00	0.04 ± 0.00	0.04 ± 0.01
**Ca**	8.94 ± 2.10 ^{a}	6.96 ± 0.53 ^{b}	7.12 ± 0.70 ^{b}	7.14 ± 0.49 ^{b}	24.30 ± 3.33 ^{b}	27.91 ± 2.02 ^{ab}	32.12 ± 2.94 ^{a}	32.00 ± 2.54 ^{a}
**Cu**	0.04 ± 0.01 ^{a}	0.02 ± 0.00 ^{b}	0.02 ± 0.00 ^{b}	0.02 ± 0.00 ^{b}	0.01 ± 0.00	0.01 ± 0.00	0.01 ± 0.00	0.01 ± 0.00
**Fe**	3.48 ± 0.67 ^{a}	1.45 ± 0.33 ^{b}	3.87 ± 0.62 ^{a}	1.85 ± 0.02 ^{b}	0.10 ± 0.01	0.09 ± 0.01	0.09 ± 0.01	0.09 ± 0.02
**K**	72.06 ± 2.46	69.04 ± 8.95	64.66 ± 1.24	62.75 ± 4.59	48.42 ± 4.04 ^{b}	55.27 ± 3.91 ^{a}	47.55 ± 2.56 ^{b}	49.82 ± 4.00 ^{b}
**Mg**	3.16 ± 0.79 ^{a}	2.90 ± 0.50 ^{a}	2.08 ± 0.14 ^{b}	2.23 ± 0.17 ^{b}	3.01 ± 0.29	3.18 ± 0.21	2.99 ± 0.26	3.33 ± 0.16
**Mn**	1.12 ± 0.13	1.15 ± 0.14	1.15 ± 0.42	1.31 ± 0.11	0.22 ± 0.03	0.23 ± 0.02	0.20 ± 0.04	0.23 ± 0.02
**Mo**	0.01 ± 0.00	0.01 ± 0.00	0.01 ± 0.00	0.01 ± 0.00	0.01 ± 0.00	0.01 ± 0.00	0.01 ± 0.00	0.01 ± 0.00
**Na**	0.71 ± 0.13	0.61 ± 0.05	0.73 ± 0.16	0.63 ± 0.05	0.20 ± 0.03	0.21 ± 0.02	0.24 ± 0.05	0.23 ± 0.03
**P**	8.31 ± 0.61 ^{a}	7.28 ± 0.50 ^{b}	8.38 ± 0.26 ^{a}	7.00 ± 0.37 ^{b}	5.66 ± 0.30	5.32 ± 0.40	5.37 ± 0.94	5.40 ± 0.30
**S**	10.94 ± 0.79	11.08 ± 0.64	10.01 ± 1.00	10.70 ± 0.44	12.97 ± 1.79	12.49 ± 0.36	11.79 ± 1.13	12.51 ± 1.48
**Zn**	0.06 ± 0.02 ^{ab}	0.04 ± 0.00 ^{c}	0.07 ± 0.00 ^{a}	0.05 ± 0.01 ^{bc}	0.032 ± 0.005 ^{a}	0.024 ± 0.002 ^{b}	0.028 ± 0.005 ^{b}	0.024 ± 0.002 ^{b}

(B)

	Roots (mg per plant)				Leaves (mg per plant)			
	Control		Low B		Control		Low B	
	-Si	+Si	-Si	+Si	-Si	+Si	-Si	+Si
**Al**	0.17 ± 0.05 ^{a}	0.06 ± 0.01 ^{b}	0.05 ± 0.01 ^{b}	0.06 ± 0.03 ^{b}	0.31 ± 0.08	0.26 ± 0.02	0.24 ± 0.07	0.29 ± 0.06
**Ca**	14.01 ± 5.93 ^{a}	10.07 ± 1.73 ^{ab}	5.26 ± 1.65 ^{b}	7.84 ± 1.65 ^{b}	179.99 ± 57.28	203.09 ± 31.92	181.36 ± 42.9	237.44 ± 46.14
**Cu**	0.06 ± 0.03 ^{a}	0.02 ± 0.004 ^{b}	0.02 ± 0.002 ^{b}	0.02 ± 0.005 ^{b}	0.04 ± 0.01 ^{a}	0.04 ± 0.01 ^{ab}	0.03 ± 0.01 ^{b}	0.04 +/- 0.01 ^{ab}
**Fe**	5.22 ± 0.65 ^{a}	2.08 ± 0.31 ^{b}	2.89 ± 1.06 ^{b}	2.03 ± 0.39 ^{b}	0.72 ± 0.11 ^{a}	0.64 ± 0.1 ^{ab}	0.49 ± 0.07 ^{b}	0.68 ± 0.16 ^{ab}
**K**	110.1 ± 19.6 ^{a}	97.67 ± 21.27 ^{a}	52.81 ± 5.74 ^{b}	68.84 ± 14.7 ^{b}	355.4 ± 85.3 ^{ab}	402.7 ± 64.6 ^{a}	268.8 ± 58.7 ^{b}	364.8 ± 37.8 ^{ab}
**Mg**	4.99 ± 2.22 ^{a}	4.12 ± 1.08 ^{a}	1.54 ± 0.49 ^{b}	2.45 ± 0.57 ^{b}	22.13 ± 5.62	23.11 ± 3.55	16.88 ± 3.75	24.74 ± 4.58
**Mn**	1.7 ± 0.27 ^{a}	1.62 ± 0.32 ^{a}	0.88 ± 0.51 ^{b}	1.52 ± 0.25 ^{a}	1.62 ± 0.44 ^{ab}	1.63 ± 0.13 ^{ab}	1.12 ± 0.33 ^{b}	1.72 ± 0.36 ^{a}
**Mo**	0.02 ± 0.01 ^{a}	0.02 ± 0 ^{a}	0.01 ± 0 ^{b}	0.01 ± 0 ^{ab}	0.09 ± 0.03	0.09 ± 0.01	0.06 ± 0.01	0.09 +/- 0.03
**Na**	1.09 ± 0.36 ^{a}	0.85 ± 0.17 ^{ab}	0.51 ± 0.09 ^{b}	0.69 ± 0.15 ^{b}	1.44 ± 0.33	1.57 ± 0.12	1.3 ± 0.25	1.66 ± 0.3
**P**	12.76 ± 3.01 ^{a}	10.23 ± 1.25 ^{b}	6.17 ± 1.76 ^{c}	7.66 ± 1.43 ^{c}	41.04 ± 6.09	38.55 ± 4.74	30.16 ± 6.8	39.86 ± 6.5
**S**	16.9 ± 4.41 ^{a}	15.62 ± 2.43 ^{ab}	7.37 ± 2.32 ^{c}	11.74 ± 2.3 ^{b}	95.33 ± 25.07	90.69 ± 10.96	66.62 ± 15.18	93.38 ± 21.58
**Zn**	0.09 ± 0.01 ^{a}	0.06 ± 0.01 ^{b}	0.06 ± 0.01 ^{b}	0.06 ± 0.01 ^{b}	0.23 ± 0.04 ^{a}	0.18 ± 0.01 ^{b}	0.15 ± 0.02 ^{b}	0.18 ± 0.03 ^{b}

Figure S1: Effect of Si application on the expression level of B transporters in rapeseed roots under short-term B deficiency; (A) Relative expression of *BnaBOR1;1a* in roots, (B) Relative expression of *BnaBOR1;1c* in roots, (C) Relative expression of *BnaBOR1;2a* in roots, (D) Relative expression of *BnaBOR1;3a* in roots, (E) Relative expression of *BnaBOR1;3c* in roots and (F) Relative expression of *BnaNIP6;1* in roots. Plants were grown in hydroponic culture under control B condition for one week (using 25 µM ^{11}B) and then for two weeks under either low or control B condition (using 0.1 µM or 25 µM ^{10}B, respectively) as well as 1.8 mM of Si supply. Roots and shoots were harvested after 0, 2, 4, 7, 10 and 15 days of treatments. The two first leaves are considered as old leaves and the other leaves developing from 7 days of B deficiency are considered as young leaves. Bars indicate mean ± SEM. Asterisks denote a significant difference according to t-test between low B and low B+Si ($p < 0.05$; n = 5).

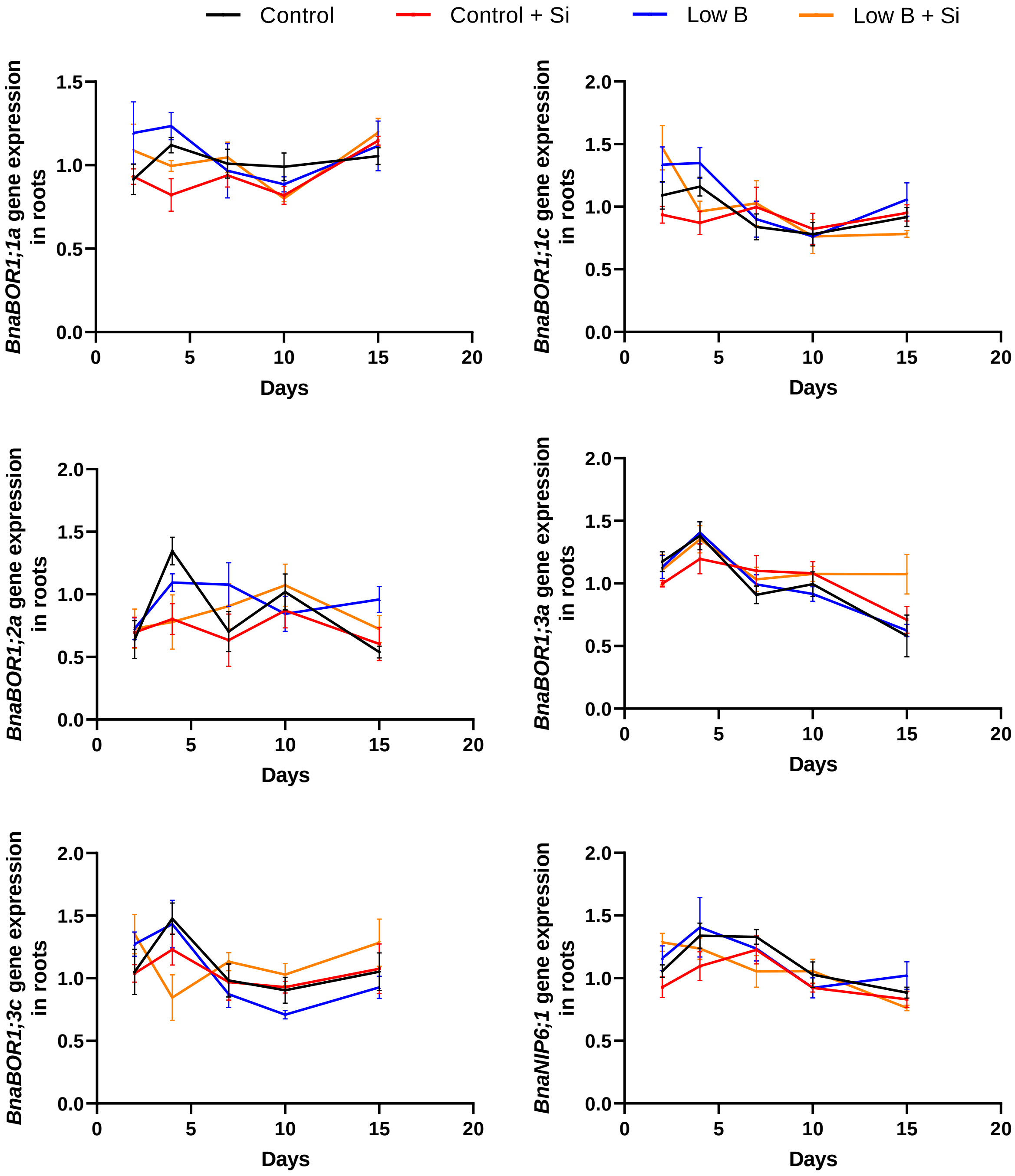

Figure S2: Effect of Si application on the expression level of B transporters in rapeseed old leaves under short-term B deficiency; (A) Relative expression of BnaBOR1;1a in old leaves, (B) Relative expression of BnaBOR1;1c in old leaves, (C) Relative expression of BnaBOR1;2a in old leaves, (D) Relative expression of BnaBOR1;3a in old leaves, (E) Relative expression of BnaBOR1;3c in old leaves and (F) Relative expression of BnaNIP6;1 in old leaves. Plants were grown in hydroponic culture under control B condition for one week (using 25 µM 11B) and then for two weeks under either low or control B condition (using 0.1 µM or 25 µM 10B, respectively) as well as 1.8 mM of Si supply. Roots and shoots were harvested after 0, 2, 4, 7, 10 and 15 days of treatments. The two first leaves are considered as old leaves and the other leaves developing from 7 days of B deficiency are considered as young leaves. Bars indicate mean ± SEM. Asterisks denote a significant difference according to t-test between low B and low B+Si ($p < 0.05$; $n = 5$).

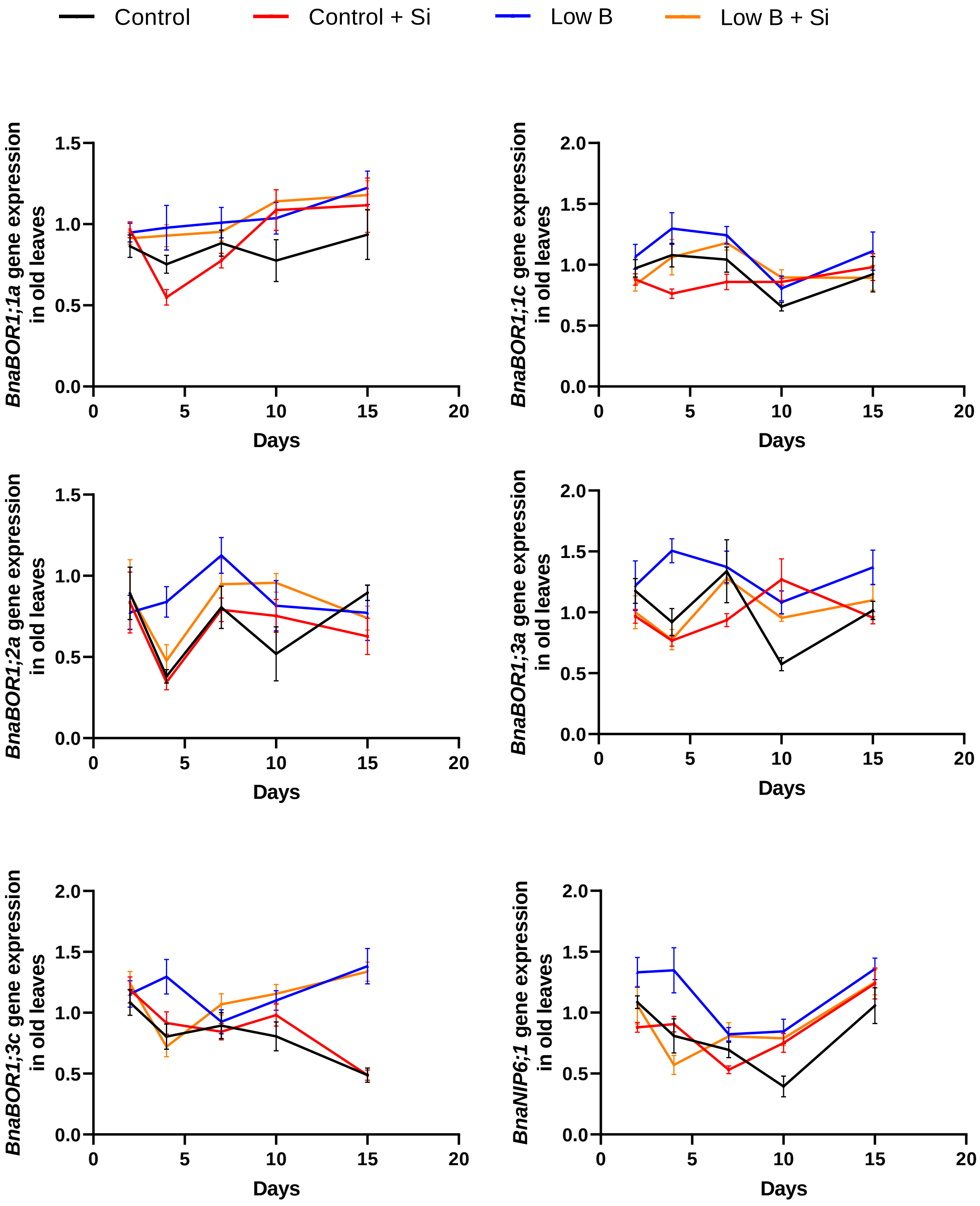

Figure S3: Effect of Si application on the expression level of B transporters in rapeseed young leaves under short-term B deficiency; (A) Relative expression of *BnaBOR1;1a* in young leaves, (B) Relative expression of *BnaBOR1;1c* in young leaves, (C) Relative expression of *BnaBOR1;2a* in young leaves, (D) Relative expression of *BnaBOR1;3a* in young leaves, (E) Relative expression of *BnaBOR1;3c* in young leaves and (F) Relative expression of *BnaNIP6;1* in young leaves. Plants were grown in hydroponic culture under control B condition for one week (using 25 µM ^{11}B) and then for two weeks under either low or control B condition (using 0.1 µM or 25 µM ^{10}B, respectively) as well as 1.8 mM of Si supply. Roots and shoots were harvested after 0, 2, 4, 7, 10 and 15 days of treatments. The two first leaves are considered as old leaves and the other leaves developing from 7 days of B deficiency are considered as young leaves. Bars indicate mean ± SEM. Asterisks denote a significant difference according to t-test between low B and low B+Si ($p < 0.05$; n = 5).

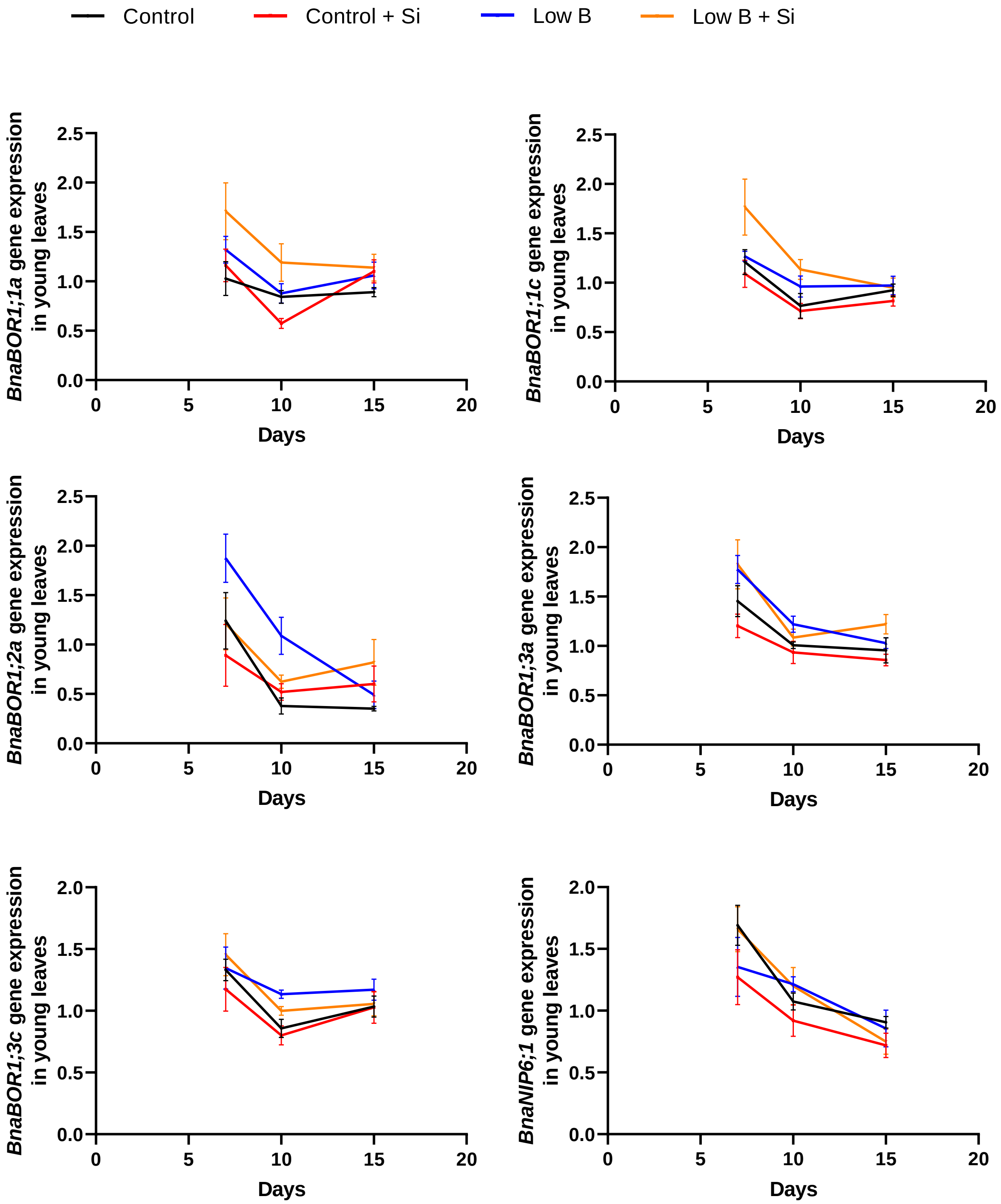

Supplement: Supplementary file 1 [file plants-12-02574-s001.zip › plants-2445355-supplementary.pdf]
